# Enteric and non-enteric adenoviruses associated with acute gastroenteritis in pediatric patients in Thailand, 2011 to 2017

**DOI:** 10.1371/journal.pone.0220263

**Published:** 2019-08-01

**Authors:** Kattareeya Kumthip, Pattara Khamrin, Hiroshi Ushijima, Niwat Maneekarn

**Affiliations:** 1 Department of Microbiology, Faculty of Medicine, Chiang Mai University, Chiang Mai, Thailand; 2 Center of Excellence in Emerging and Re-emerging Diarrheal Viruses, Chiang Mai University, Chiang Mai, Thailand; 3 Department of Developmental Medical Sciences, School of International Health, Graduate School of Medicine, The University of Tokyo, Tokyo, Japan; 4 Division of Microbiology, Department of Pathology and Microbiology, Nihon University School of Medicine, Tokyo, Japan; Harvard Medical School, UNITED STATES

## Abstract

Human adenovirus (HAdV) is known to be a common cause of diarrhea in children worldwide. Infection with adenovirus is responsible for 2–10% of diarrheic cases. To increase a better understanding of the prevalence and epidemiology of HAdV infection, a large scale and long-term study was needed. We implemented a multi-year molecular detection and characterization study of HAdV in association with acute gastroenteritis in Chiang Mai, Thailand from 2011 to 2017. Out of 2,312 patients, HAdV was detected in 165 cases (7.2%). The positive rate for HAdV infection was highest in children of 1 and 2 years of age compared to other age groups. HAdV subgroup C (40.6%) was the most prevalent, followed by subgroups F (28.5%), B (20.6%), A and D (4.8% each), and E (0.6%). Of these, HAdV-F41 (22.4%), HAdV-C2 (18.2%), HAdV-B3 (15.2%), and HAdV-C1 (13.3%) were the most common genotypes detected. HAdV infection occurred throughout the year with a higher detection rate between May and July. In conclusion, our study demonstrated the infection rate, seasonal distribution and genotype diversity of HAdV infection in children with diarrhea in Chiang Mai, Thailand over a period of 7 year. Not only enteric adenovirus (F40 and F41) but also non-enteric adenovirus (B3, C1, C2) may play an important role in gastroenteritis in this area. The information will be beneficial for the prevention and control of HAdV outbreaks in the future.

## Introduction

Acute gastroenteritis or diarrhea is a major cause of morbidity, mortality and is still a significant public health problem of infants and young children worldwide. Adenovirus has been recognized as one of the important pathogens responsible for diarrhea. Adenoviruses belong to the family *Adenoviridae*. The virus is a spherical and non-enveloped particle with a diameter of 70–90 nm. The genome of this virus is a double-stranded DNA with 26–45 kb in length encapsulated within an icosahedral capsid. Several viruses in the *Adenoviridae* family can cause diseases in humans and many other animals [1]. To date, five genera of the *Adenoviridae* family have been classified including *Mastadenovirus*, *Aviadenovirus*, *Atadenovirus*, *Siadenovirus*, and *Ichtadenovirus*. The Mastadenovirus infects mammalian species whereas the Aviadenovirus infects avian species. The Atadenovirus and Siadenovirus infect a wide range of hosts while the Ichtadenovirus, which comprises only one member, infects fish (white sturgeon adenovirus 1) [2].

The adenovirus that can infect humans, the so called human adenovirus (HAdV), was first discovered in 1953 by Rowe and colleagues [3]. The first virus identified was isolated from adenoidal tissue of children and this virus was named “adenovirus”. Adenovirus was then classified as a member of the Mastadenovirus in 1999 by the International Committee of Taxonomy of Viruses. Currently, HAdV is classified into 9 subgroups (A to I) and within each subgroup 90 genotypes have been identified [2,4]. Adenovirus infection leads to pathogenesis of several systems in human. All subgroups of adenovirus infect the respiratory tract while subgroup F of serotypes 40 and 41 infect the gastrointestinal tract. Adenovirus serotypes 40 and 41 are the most common cause (5–20%) of acute gastroenteritis in young children [5–8]. Adenovirus can be transmitted by inhalation, direct contact with small droplets, or the fecal-oral route. It has been reported that adenovirus is a causative agent of several outbreaks in communities such as schools, nurseries and military camps, the virus being able to contaminate environmental sources including food and water [9–12]. Adenovirus infected patients normally present clinical symptoms such as diarrhea, vomiting and complications of the respiratory system. Adenovirus infection rarely causes serious illness or death. However, infants and immunocompromised hosts, or patients who have respiratory or cardiac diseases, are at higher risk of developing a severe illness from an adenovirus infection [13,14]. Epidemiological studies of HAdV conducted in several countries around the world including Bangladesh, Brazil, Korea, China, and Japan have demonstrated that the prevalence of HAdV in diarrheic cases varied from 2–10% and the virus predominantly infected young children less than 2 years of age [9,15–18]. In Thailand, knowledge regarding the prevalence of adenovirus infection in diarrheic children is not well documented. Only a few studies have reported the detection of HAdV in patients with diarrhea [16,19,20] and those studies have not been conducted continuously. To increase a better understanding of the prevalence and epidemiology of HAdV infection with a large scale and long-term study, we investigated a multi-year molecular epidemiology and characterization of HAdV genotypes in association with acute gastroenteritis in Chiang Mai, Thailand during the period 2011 to 2017.

## Materials and methods

### Patients and specimen collection

The study was approved by the Research Ethics Committee of the Faculty of Medicine, Chiang Mai University (MIC-2560-04649). The written informed consent form was obtained from parents before samples were collected from their children. Fecal specimens were collected from children less than 5 years of age who were admitted to hospitals with acute gastroenteritis (≥ 3 loose or looser than normal stools in a 24-hour period but excluded those with pus or blood) between January 2011 and December 2017. These fecal samples were collected from children who had been admitted to several major hospitals in Chiang Mai Province, Thailand, including Maharaj Nakorn Chiang Mai Hospital, Sriphat Medical Center, Nakornping Hospital, and Sanpatong Hospital. All the samples were kept frozen at -20°C until investigation.

### Sample preparation

Fecal specimens were suspended in phosphate buffered saline (pH 7.4) as 10% suspensions (w/v). Stool suspensions were then centrifuged at 5,000 rpm for 5 minutes and viral nucleic acid was extracted from 200 μl of the supernatant using a Geneaid Viral Nucleic Acid Extraction Kit II (Geneaid, Taipei, Taiwan) according to the manufacturer’s protocol. The viral genomic DNA was either subjected immediately to PCR assay or stored at -70°C until investigation.

### Human adenovirus detection

The presence of human adenovirus in stool samples was detected by PCR amplification of the hexon gene of HAdV using primers Ad1 (5’-TTCCCCATGGCTCAYAACAC-3’) and Ad2 (5’-CCCTGGTAKCCRATRTTGTA-3’) as described previously [21]. The PCR amplification was performed using a GoTaq DNA polymerase (Promega, Wisconsin, USA) in accordance with the manufacturer’s protocol. The PCR amplification was carried out under the following conditions: 3 minutes at 94°C, 1 minute at 94°C, 1 minute at 50°C and 1 minute at 72°C for 35 cycles, followed by 72°C for 10 minutes. The PCR products were subjected to 1.5% agarose gel electrophoresis, stained with nucleic acid staining solution (RedSafe, INtRON Biotechonology, South Korea) and visualized under a UV-transilluminator with the expected PCR product size of 482 bp.

### Phylogenetic analysis

The amplicons were purified using a Gel/PCR DNA Fragment Extraction Kit (Geneaid, Taiwan) in accordance with the manufacturer’s protocol and direct sequenced using the Ad1 forward primer and Big Dye Terminator Cycle Sequencing Kit (Thermo Fisher Scientific, Massachusetts, USA), and analyzed on an automated DNA sequencer ABI3100 Applied Biosystems (Applied Biosystems, California, USA). The obtained nucleotide sequences of adenovirus detected in this study were analyzed in comparison with those of the reference virus strains available in the GenBank database. Phylogenetic analysis of the partial hexon gene was performed using MEGA X with 1000 bootstrap replicates complemented with BioEdit and Clustal X softwares. The nucleotide sequences of human adenovirus described in this study are available in the GenBank database under the accession numbers MK674616 to MK674780.

### Statistical analysis

Statistical analysis was performed using SPSS software version 22.0. The Chi-square test was used to measure the differences in frequencies of virus infection between age groups and used to determine the significance of HAdV infection rates between gender/ months. A *p-*value of < 0.05 was considered to be statistically significant.

## Results

### Detection rate of human adenovirus infection

**Fig 1:**
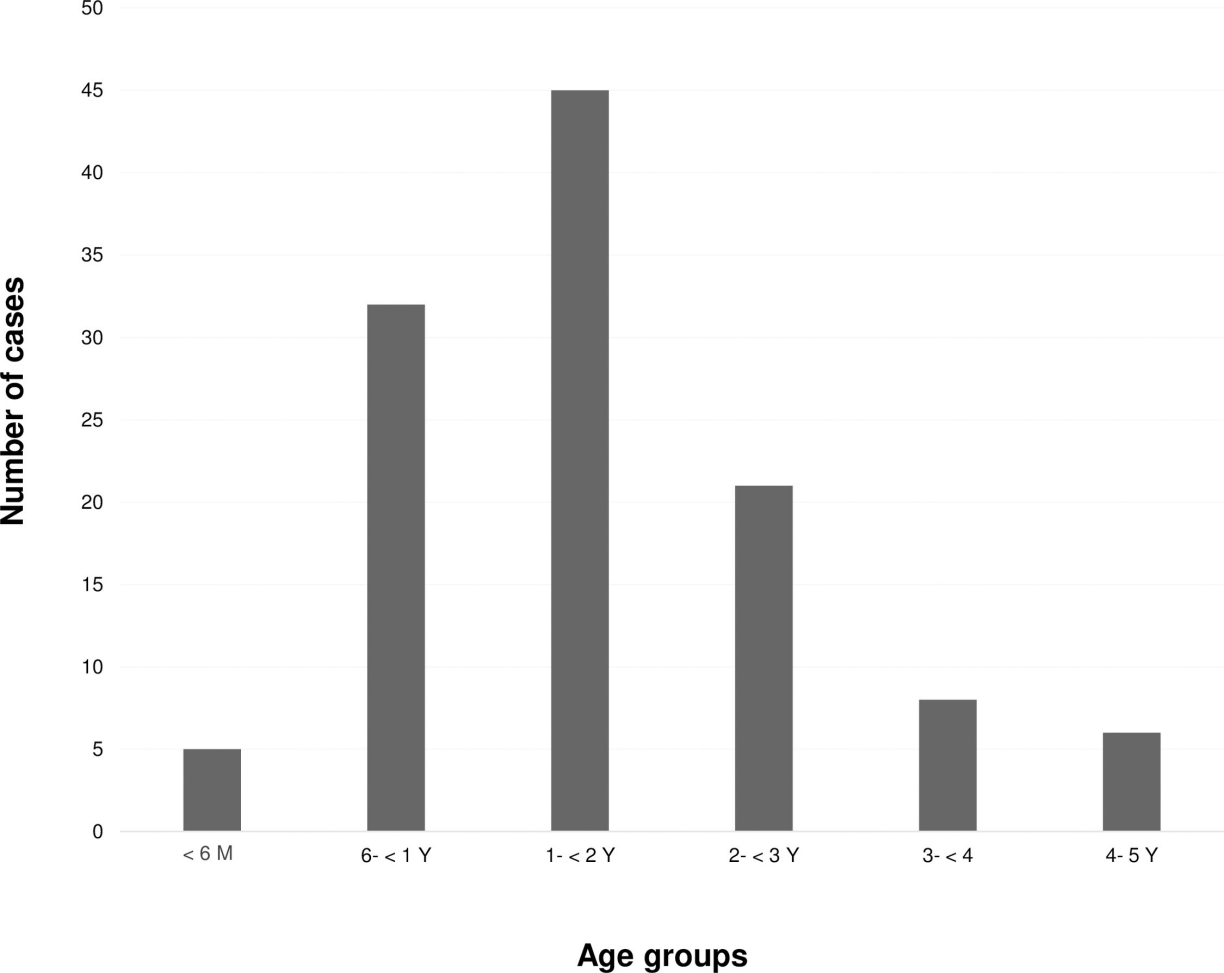
Detection of human adenovirus in different age groups of patients.

During the study period of 7 years, HAdV was detected in 165 (7.2%) out of 2,312 fecal specimens of the hospitalized cases. The prevalence of HAdV infection stratified over the 7 years were: 3.6% in 2011, 5.9% in 2012, 8.2% in 2013 and 2014, 11.6% in 2015, 7.3% in 2016, and 4.7% in 2017 (Table 1). Out of 2,312 specimens, 1,334 were collected from boys and 972 from girls (gender information of 6 children were not available). The mean age of HAdV-infected patients was 1.7 years (min = 4 months and max = 5 years). Among the 165 HAdV positive cases, 54% (88/163) were male and 46% (75/163) were female (1.2:1) while the gender information of 2 children were not available. The detection rates of HAdV infection found in male and female groups were quite similar at 6.6% (88/1,334) and 7.7% (75/972), respectively and were not statistically significantly different (*p =* 0.300). It was found that children of between 1 and 2 years of age represented the majority of infected patients at 38.5%, followed by children at the age of 6 months to <1 year, 2 to <3 years, 3 to <4 years, 4 to 5 years, and less than 6 months at 27.4%, 17.9%, 6.8%, 5.1%, 4.3%, respectively (Fig 1). HAdV infection was significantly higher in patients with the age of 1 to 2 years than other age groups (*p <* 0.001).

**Table 1:** Detection rates of HAdV infection in children with acute gastroenteritis in Chiang Mai, Thailand from 2011 to 2017.

Year of study	No. of samples tested	No. of AdV positive (%)	Total samples / positive samples (%)	
			Male^a^	Female^a^
2011	302	11 (3.6)	170/5 (2.9)	131/6 (4.6)
2012	341	20 (5.9)	194/9 (4.6)	146/10 (6.9)
2013	280	23 (8.2)	159/16 (10.0)	117/6 (5.1)
2014	268	22 (8.2)	145/7 (4.8)	123/15 (12.2)
2015	335	39 (11.6)	200/28 (14.0)	135/11 (8.2)
2016	508	37 (7.3)	295/18 (6.1)	213/19 (8.9)
2017	278	13 (4.7)	171/5 (2.9)	107/8 (7.5)
7 years	2,312	165 (7.2)	1,334/88 (6.6)	972/75 (7.7)

^a^ Gender information of six patients not available

### Monthly distribution of human adenovirus infection

**Fig 2:**
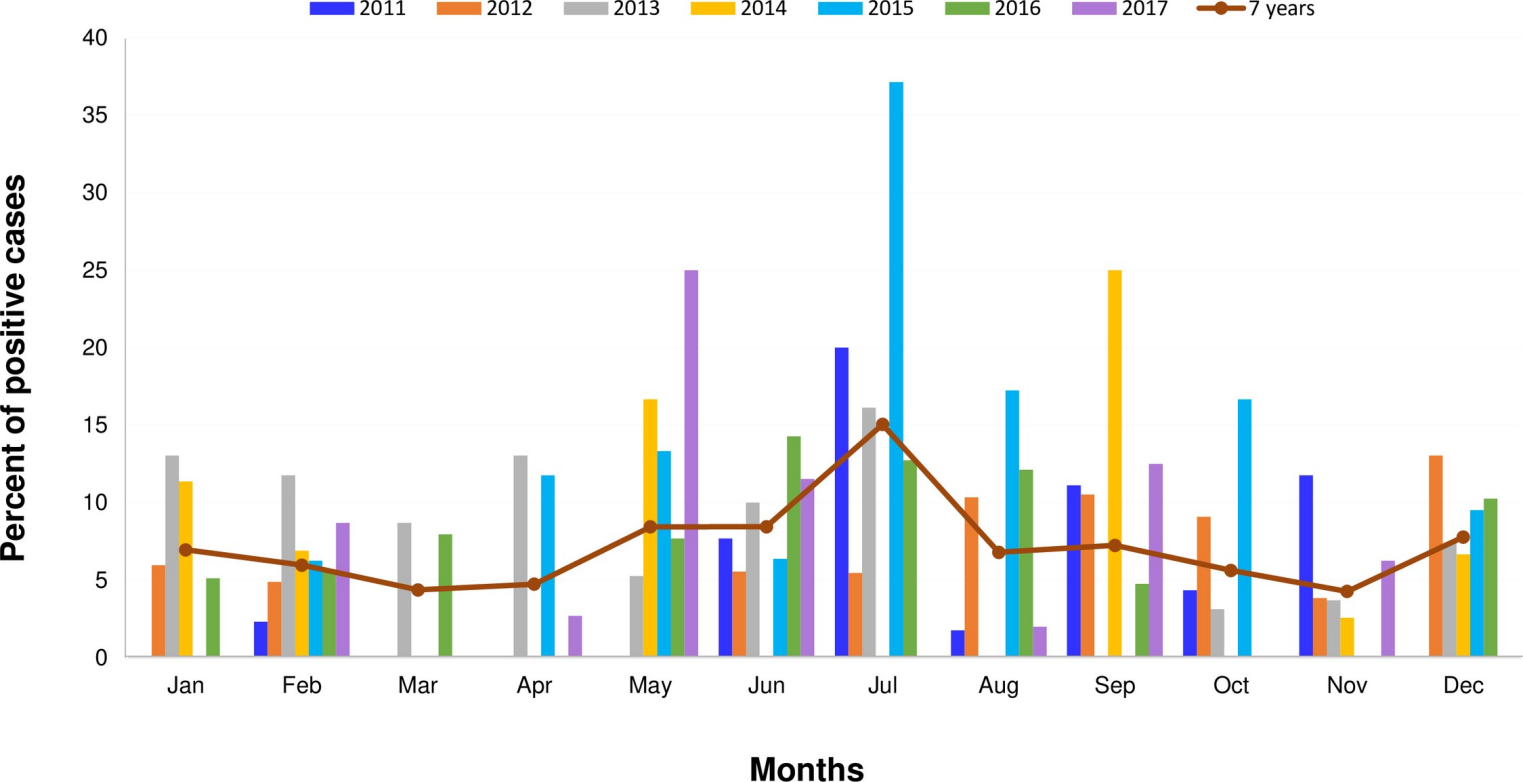
Monthly distribution of adenovirus infections in Thai patients with acute gastroenteritis from 2011 to 2017.

The seasonal distribution of HAdV infection from January 2011 to December 2017 is presented in Fig 2. The data revealed that HAdV infection occurred all year round with a peak period from May to July (8.9–15%) which corresponded to the rainy season in Thailand (*p =* 0.005). Monthly distribution of HAdV-positive cases, as analyzed by year, indicated that the highest rates of HAdV detection in 2011 was in July (20%), 2012 in December (13%), 2013 in July (16.1%), 2014 in September (25%), 2015 in July (37.1%), 2016 in June (14.3%), and 2017 in May (25%).

### Distribution of human adenovirus genotype

**Fig 3:**
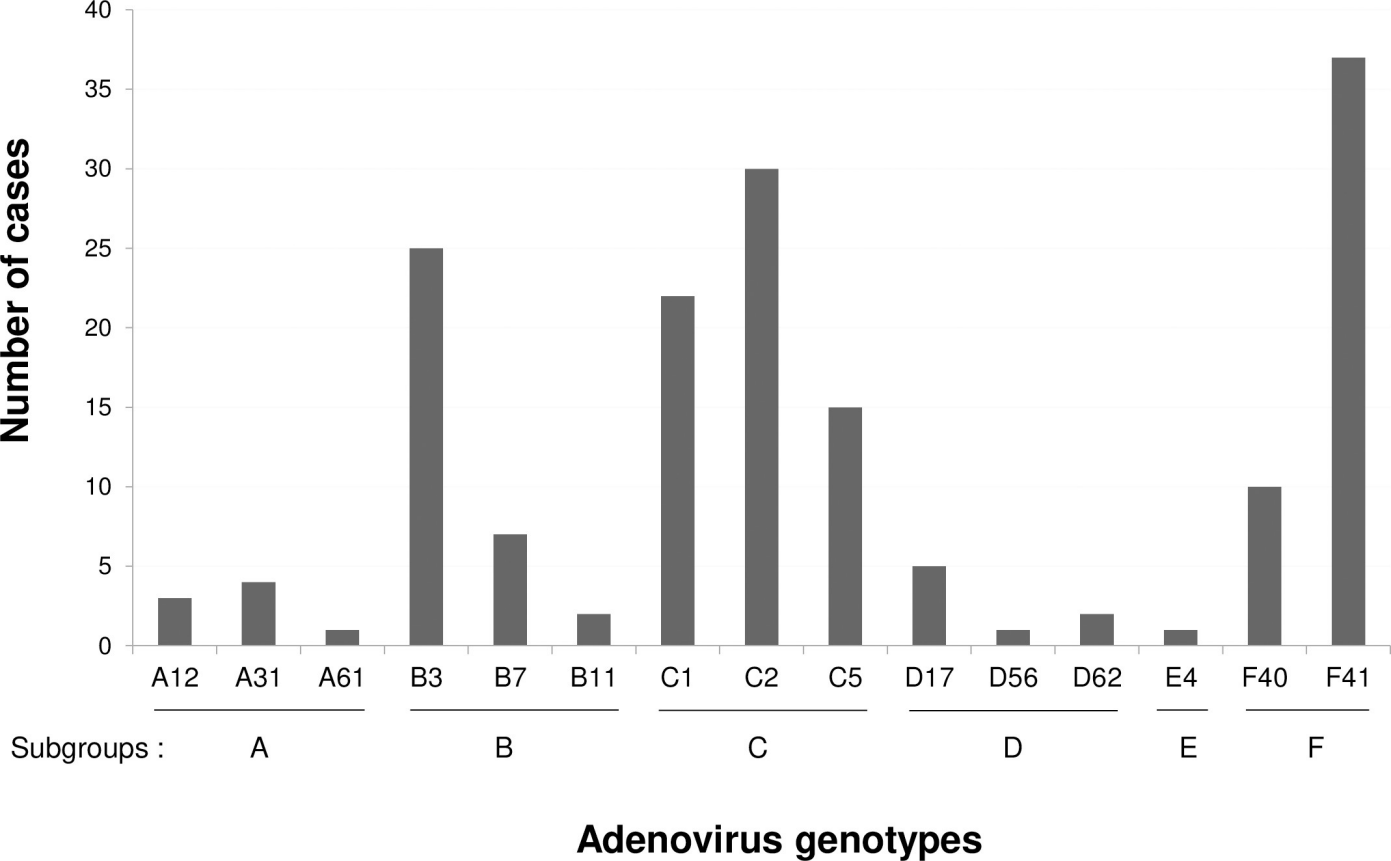
Distribution of human adenovirus genotypes in patients with acute diarrhea in Chiang Mai, Thailand during the study period of seven years from 2011 to 2017.

Over the 7-year period of study, 6 subgroups (A to F) of adenovirus were detected and 15 different genotypes were identified (Fig 3). Subgroups C, F, and B had the highest prevalence at 40.6%, 28.5%, and 20.6%, respectively. Of the 15 genotypes, HAdV-F41 (22.4%) was the most predominant genotype, followed by HAdV-C2 (18.2%), HAdV-B3 (15.2%), HAdV-C1 (13.3%), HAdV-C5 (9.1%), HAdV-F40 (6.1%), HAdV-B7 (4.2%), HAdV-D17 (3.0%), HAdV-A31 (2.4%), HAdV-A12 (1.8%), HAdV-B11 and HAdV-D62 (1.2% each), HAdV-A61, HAdV-D56, and HAdV-E4 (0.6% each). Interestingly, the HAdV-F41 and HAdV-C2 genotypes were detected in ever year of the study period while HAdV-C1 was detected from 2012 to 2017. The HAdV-C5 genotype was also detected in ever year of the study period from 2011 to 2017, except for 2016. The HAdV-B3 was detected in 2011 for the first time and disappeared in 2012, and then it was detected again in 2013 and continued to 2017 (Table 2).

**Table 2:** Distribution of HAdV genotypes in children with diarrhea in Chiang Mai, Thailand during 2011 to 2017.

Years	HAdV genotypes														
	A12	A31	A61	B3	B7	B11	C1	C2	C5	D17	D56	D62	E4	F40	F41
2011 (n = 11)	1	-	-	3	2	-	-	1	1	1	-	-	-	-	2
2012 (n = 20)	1	3	-	-	-	-	1	3	1	2	1	-	-	3	5
2013 (n = 23)	-	-	-	2	1	1	4	4	1	1	-	2	-	2	5
2014 (n = 22)	-	-	1	2	-	-	4	8	6	-	-	-	-	-	1
2015 (n = 39)	1	1		4	-	-	7	8	4	1	-	-	1	1	11
2016 (n = 37)	-	-	-	12	3	1	3	5	-	-	-	-	-	2	11
2017 (n = 13)	-	-	-	2	1	-	3	1	2	-	-	-	-	2	2
7 years (n = 165)	3	4	1	25	7	2	22	30	15	5	1	2	1	10	37

### Distribution of human adenovirus genotypes in different age groups

**Fig 4:**
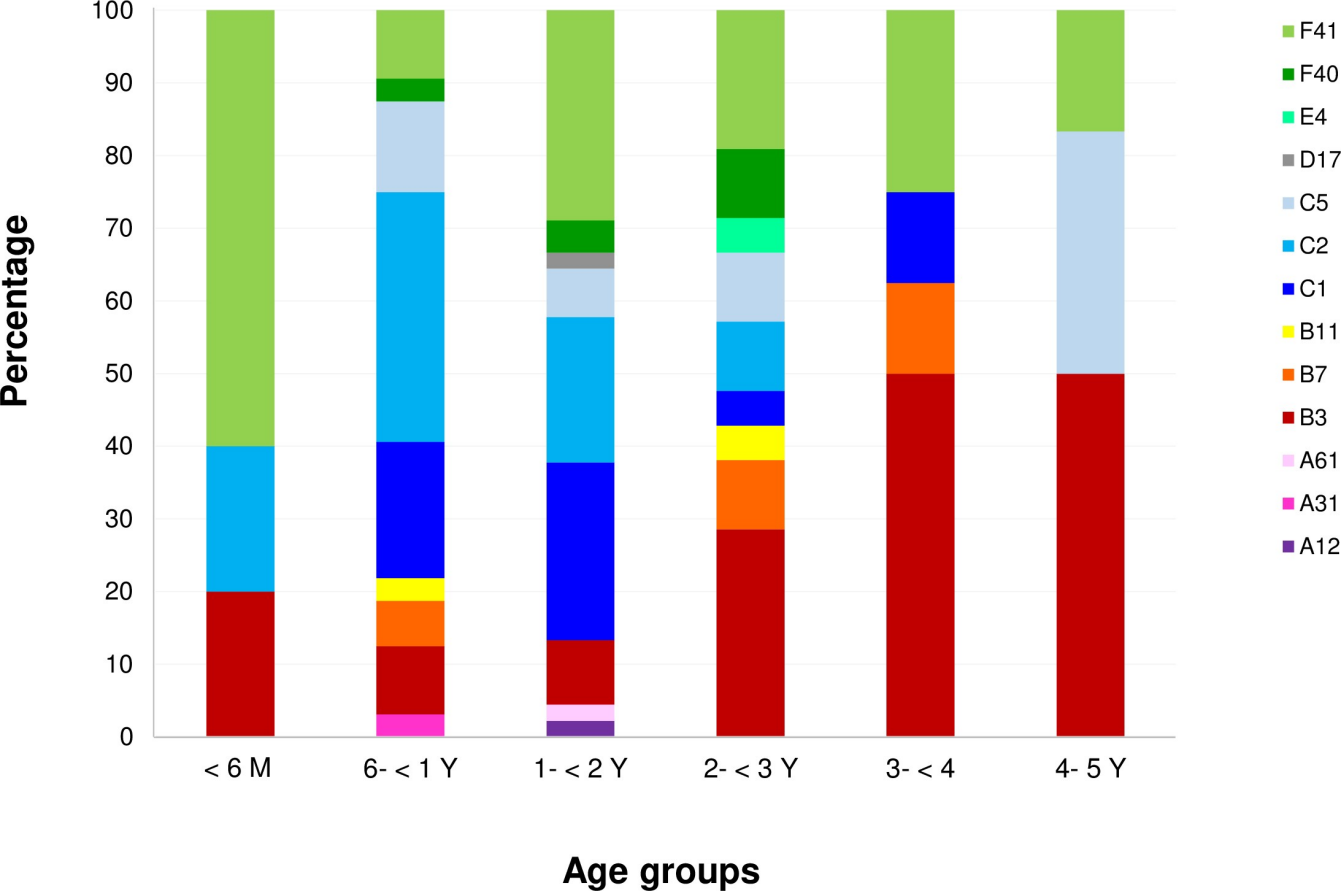
Distribution of human adenovirus genotypes among children of different age groups.

Since this study found a diverse HAdV genotypes in children with diarrhea, we further investigated the distribution of the virus genotypes in the patients of different age groups. The data showed that the patients aged from 6 months to 3 years were infected with a wide variety of HAdV genotypes (9 different genotypes) while children in other age groups were infected with only 3 or 4 genotypes (Fig 4). It should be noted that the HAdV-F41 and HAdV-B3 were detected in all age groups of the patients with the highest proportion of cases, 22.2% and 17.9%, respectively. On the other hand, the HAdV-C1, C2 and C5 genotypes were frequently detected in children with the age of 6 months to 3 years.

### Phylogenetic analysis of the hexon gene of human adenoviruses

**Fig 5:**
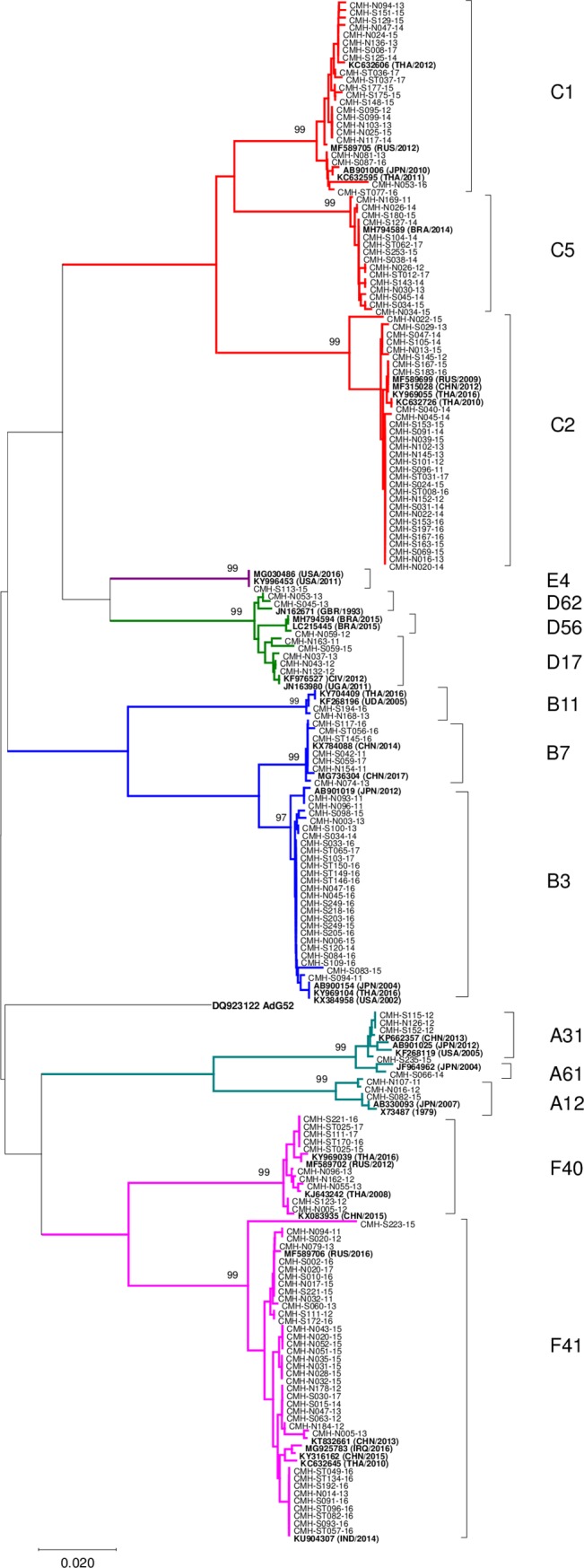
Phylogenetic tree of partial nucleotide sequences (429 bp) of the hexon gene. Human adenovirus strains detected in Thailand between 2011 and 2017 (165 sequences) and other 41 adenovirus reference strains (bold) are included.

A phylogenetic tree of the partial hexon gene was constructed to identify HAdV genotypes and to determine the genetic relationship between the 165 HAdV strains detected in this study and with other HAdV strains previously reported worldwide. The phylogenetic tree revealed that HAdV strains were classified into distinctive genotypic clusters (Fig 5). The overall nucleotide sequence identities of HAdV strains detected in this study were highly similar (96.7–100%) to each other and to those of the HAdV strains reported previously from Thailand, Japan, China, India, Russia, Brazil, Uganda, Iraq, United Kingdom, and United States. The HAdV-F41 viruses detected in this study were closely related to the HAdV strains detected previously in China, India, Russia and Thailand with a 93.7–99.7% nucleotide sequence identity. The HAdV-C2 strains displayed a high degree of similarity (99.3–100%) of nucleotide sequence to the HAdV-C2 strains reported from Thailand, China and Russia. The HAdV-C1 strains showed a 97.4–99.7% nucleotide sequence identity to the viruses reported from Thailand, Japan and Russia while the HAdV-C1 strains were closely related to an HAdV-C5 strain detected in Brazil. The HAdV-B3 strains were closely related to the HAdV-b3 strains reported from Thailand, Japan, India, and United States.

## Discussion

Human adenovirus is known to be a common cause of diarrhea in children after rotaviruses and caliciviruses. Infection with adenovirus is responsible for 1–10% of diarrheic cases [22]. The overall HAdV infection rate of children with diarrhea observed in this study was 7.2% (a range of 3.6–11.6%) which is similar to those reported from other countries, for example, Tanzania (3.5%) [23], Japan (4.8–7.9%) [18,24], Korea (5.5%) [25], China (9.8%) [17], India (11.8%) [5], and also reports from other regions in our country, Bangkok and Khon Kaen (5.8%) [16]. HAdV was detected at lower rates in 2011 (3.6%) and 2012 (5.9%) but the prevalence increased from 2013 to 2015 with the highest rate in 2015 (11.6%). The high prevalence of HAdV infection in 2015 may be due to the co-circulation of both enteric HAdV-F41 and non-enteric adenovirus subgroup C in this population of patients. Notably, we found that the highest frequencies of HAdV-F41 and adenovirus subgroup C were mainly in fecal specimens collected in July 2015.

In addition, we also compared the distribution of adenovirus infection in patients of different ages and genders. Our results suggested that there was no different between the rate of adenovirus infection in male and female groups (ratio of 1.2:1) which is in line with the findings from previous studies [16,17]. The incidence of HAdV infection was high in children at the age group 6 months to 2 years. These results are also comparable to previous reports [16,17,26].

Enteric adenovirus serotypes 40 and 41 have been recognized as a common pathogen associated with acute gastroenteritis. Previous epidemiological studies reported that HAdV-40 and 41 were predominant genotypes detected in patients with gastroenteritis with the rate ranging from 25 to 50% [6,16,17,23,25]. In accordance with previous studies, our study in children with diarrhea indicated that HAdV-F40 and F41 accounted for 28.5% of cases (22.4% for HAdV-F41 and 6.1% for HAdV-F40). Interestingly, the present study found that non-enteric adenovirus subgroup C (C1, C2, C5) unexpectedly became the first leading subgroup detected in diarrheic children with a prevalence of 40.6% (67/165) while enteric adenovirus subgroup F (F40 and F41) and other non-enteric adenovirus subgroup B (B3, B7, B11) ranked second and third with rates of 28.5% (47/165) and 20.6% (34/165), respectively. Non-enteric adenoviruses have also been reported to associate with gastroenteritis. For example, HAdV-B3 has been demonstrated to cause diarrhea in infants and children [6]. HAdV-C2 and C5 have also been reported to be common genotypes detected in patients with acute gastroenteritis [16]. This study demonstrated that non-enteric adenovirus genotypes C2 (18.2%), B3 (15.2%), C1 (13.3%), and C5 (9.1%) were frequently detected in our study population suggesting the potential role of non-enteric adenovirus in diarrheal disease.

Acute gastroenteritis is caused by a number of enteric viruses. In this study, we also tested for other gastroenteritis viruses, including rotavirus, norovirus, astrovirus, enterovirus, human parechovirus, human bocavirus, Aichi virus, saffold virus, and cosavirus. We found that 44.8% of HAdV-positive samples were co-infected with other enteric viruses while 55.2% of cases were solely infected with HAdV. In the cases of sole infection by HAdV, 33% and 67% of cases were caused by enteric and non-enteric adenoviruses, respectively. This data indicated that both enteric and non-enteric adenoviruses are associated with diarrhea. However, we could not exclude the possibility that some diarrheic cases may be caused by bacteria or other pathogens which have not been investigated in the present study.

Seasonal distribution of adenovirus infection remains a controversial topic. The seasonal peak of adenovirus infections varied depending on the geographical region and weather pattern of the country. In this study, we did not observe a consistent seasonal pattern of adenovirus gastroenteritis infections. However, the highest rates of HAdV infection during the 7-year study period were observed in the period May to July, our findings are similar to other studies reported previously in Thailand [16,20], Bangladesh [15], and India [5]. In 2014, HAdV-C1, C2, and C5 were the predominant genotype. In 2015, both HAdV subgroup C (C1, C2, C5) and HAdV-F41 were co-circulating in patients with diarrhea. In 2016, HAdV subgroup B (B3, B7, B11) emerged and was co-detected with HAdV-F41 instead of subgroup C. Our results demonstrated the wide variation of the HAdV genotype and annual distribution of HAdV infection. The data provide more epidemiologic information of HAdV and may be useful for the prevention and control of HAdV infection in young children and for future vaccine development.

In conclusion, this study demonstrates the prevalence and high diversity of HAdV in pediatric patients hospitalized with acute gastroenteritis over the period of 7-year study and implies that not only enteric HAdV-F40 and F41, but also non-enteric HAdV-B3, C1, C2, and C5 may play an important role in acute gastroenteritis in Thai children.
